# Cross-sectional analysis of a large cohort with X-linked Charcot-Marie-Tooth disease (CMTX1)

**DOI:** 10.1212/WNL.0000000000004296

**Published:** 2017-08-29

**Authors:** Francis B. Panosyan, Matilde Laura, Alexander M. Rossor, Chiara Pisciotta, Giuseppe Piscosquito, Joshua Burns, Jun Li, Sabrina W. Yum, Richard A. Lewis, John Day, Rita Horvath, David N. Herrmann, Michael E. Shy, Davide Pareyson, Mary M. Reilly, Steven S. Scherer, For the Inherited Neuropathies Consortium—Rare Diseases Clinical Research Network (INC-RDCRN)

**Affiliations:** From the Department of Neurology (F.B.P., D.N.H.), University of Rochester Medical Center, NY; MRC Centre for Neuromuscular Diseases (M.L., A.M.R., M.M.R.), UCL Institute of Neurology, UK; Department of Neurology (C.P., D.P.), Carlo Besta Neurological Institute, Milan, Italy; Department of Neurosciences (G.P.), Institute of Telese Terme (BN), Italy; Children's Hospital at Westmead (J.B.), University of Sydney, Australia; Department of Neurology (J.L.), Vanderbilt University, Nashville, TN; Neuromuscular Program (S.W.Y.), Children's Hospital of Philadelphia, PA; Department of Neurology (R.A.L.), Cedars-Sinai Medical Center, Los Angeles, CA; Department of Neurology (J.D.), Stanford University, CA; Institute of Genetic Medicine (R.H.), Newcastle University, UK; Department of Neurology (M.E.S.), University of Iowa Hospitals and Clinics; and Department of Neurology (S.S.S.), University of Pennsylvania, Philadelphia.

## Abstract

**Objective:**

To extend the phenotypic description of Charcot-Marie-Tooth disease (CMTX1) and to draw new genotype-phenotype relationships.

**Methods:**

Mutations in *GJB1* cause the main X-linked form of CMTX (CMTX1). We report cross-sectional data from 160 patients (from 120 different families, with 89 different mutations) seen at the Inherited Neuropathies Consortium centers.

**Results:**

We evaluated 87 males who had a mean age of 41 years (range 10–78 years) and 73 females who had a mean age of 46 years (range 15–84 years). Sensory-motor polyneuropathy affects both sexes, more severely in males than in females, and there was a strong correlation between age and disease burden in males but not in females. Compared with females, males had more severe reduction in motor and sensory neurophysiology parameters. In contrast to females, the radial nerve sensory response in older males tended to be more severely affected compared with younger males. Median and ulnar nerve motor amplitudes were also more severely affected in older males, whereas ulnar nerve motor potentials tended to be more affected in older females. Conversely, there were no statistical differences between the sexes in other features of the disease, such as problems with balance and hand dexterity.

**Conclusions:**

In the absence of a phenotypic correlation with specific *GJB1* mutations, sex-specific distinctions and clinically relevant attributes need to be incorporated into the measurements for clinical trials in people with CMTX1.

**ClinicalTrials.gov identifier:**

NCT01193075.

Charcot-Marie-Tooth disease (CMT) is a group of inherited neuropathies characterized by a nonsyndromic, chronic, motor, and sensory polyneuropathy. CMTX1 is X-linked; it is the second most common subtype, after CMT1A,^1^ and is caused by mutations in the *GJB1*.^2^ *GJB1* encodes connexin32 (Cx32), an intrinsic membrane protein of the gap junction gene family.^3^ The function of Cx32 that may be relevant to neuropathy is that it forms gap junctions between the layers of the Schwann cell myelin sheath.^4^

Over 400 *GJB1* mutations have been identified, affecting all domains of the Cx32 protein (figure 1) (hihg.med.miami.edu/code/http/cmt/public_html/index.html#/). The majority are missense mutations, but other mutations have been described; including in the noncoding region.^5^ With some exceptions, mutations that result in an altered amino acid sequence cause CMTX1,^6^ and most mutations likely cause loss of function.^7^ CMTX1 is considered a dominant disease because most female carriers are affected, albeit less so than males.^8–13^ The presumption is that *GJB1* is subjected to random X-inactivation, so that myelinating Schwann cells in affected women have mosaic expression of the mutant and wild-type *GJB1*; this has been directly shown in mice.^14^

**Figure 1:**
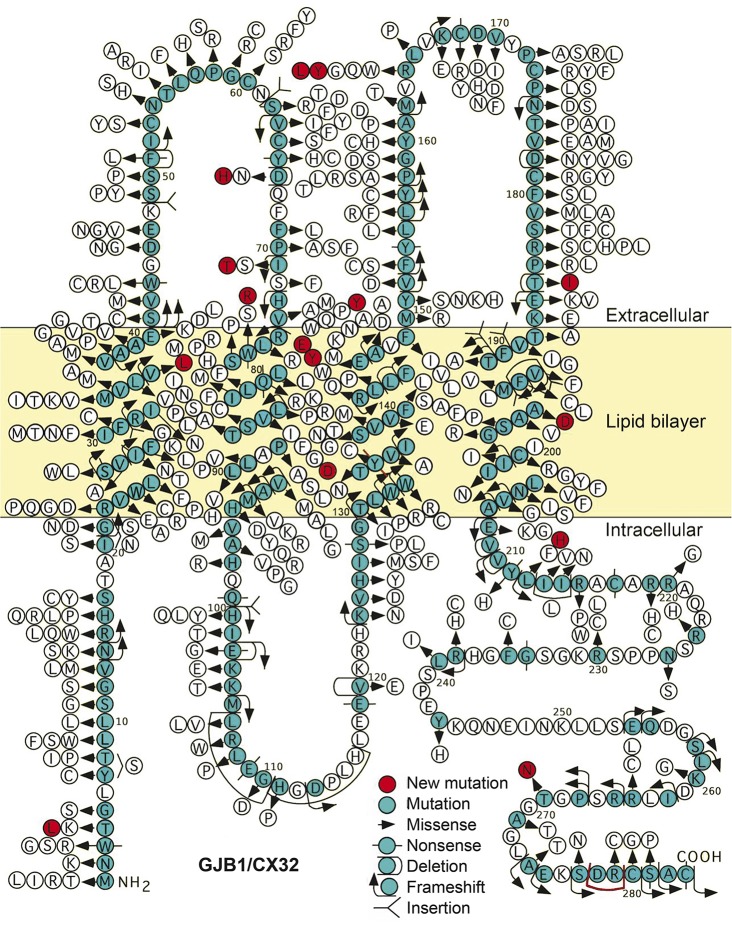
Amino acid sequence and reported *GJB1* mutations Schematic diagram showing the predicted amino acid sequence and all 420 reported *GJB1* mutations (Inherited Neuropathies Consortium variant Browser [hihg.med.miami.edu/code/http/cmt/public_html/index.html#/]). Previously unreported mutations reported are shown in red.

The objective of this study was to extend the phenotypic description of Charcot-Marie-Tooth disease (CMTX1) and to draw new genotype-phenotype relationships. We report on a large cohort of patients with CMTX1 who were seen at centers affiliated with the Inherited Neuropathies Consortium (INC).

## METHODS

### Standard protocol approvals, registrations, and patient consents.

The NIH-RDCRN and institutional review boards in the participating centers approved all aspects of this study. This trial was registered at clinicaltrials.gov (ID number: NCT01193075). As directed by the institutional review board, all registry and survey participants received and reviewed a detailed information letter and provided consent before their involvement in this research.

### Study design, setting, and patient population.

Patients were evaluated at one of the 17 INC centers between 2009 and 2015, and at Wayne State University between 1996 and 2009. The diagnosis of CMTX1 was established by sequencing *GJB1* either in the patients or in a first- or second-degree relative. All patients with CMTX1 with *GJB1* sequencing were included in this study. History, neurologic examination, and nerve conduction studies were collected.

### Clinical and neurophysiologic data acquisition.

The Charcot-Marie-Tooth disease neuropathy score (CMTNS) version 1 and/or 2 was used; this is a simple, reliable, and validated standardized assessment tool in adults with CMT.^15,16^ CMTNS is a composite score based on the history of the symptoms, neurologic examination, and clinical neurophysiology. A CMTNS score of 0–10 is considered mild impairment, 11–20 is considered moderate impairment, and ≥21 is considered severe impairment. The Charcot-Marie-Tooth disease examination score (CMTES) is the sum of the symptoms and signs portions from the CMTNS without the neurophysiologic data. CMTES was used for patients who did not undergo neurophysiologic testing or calculated from CMTNS for patients with neurophysiologic testing. As in the CMTNS, the higher the CMTES, the greater the disability. Clinical neurophysiologic studies were performed with the temperature maintained at or above 32°C in the hands and feet. Nerve conduction studies were performed using surface electrodes by standard techniques.

### Statistical analysis.

Descriptive statistics were used to analyze patient characteristics and all available data related to history, clinical examination, and neurophysiologic testing during the patient encounter. Our complete-case analyses used missing data at random assumptions. Two-tailed tests were used when applicable. A *p* value of <0.05 was considered statistically significant. The Fisher exact test, Mann-Whitney *U* test, Spearman ρ, analysis of covariance (ANCOVA), and analysis of variance were calculated using StatPlus Professional version 5.9.8.5.

## RESULTS

We evaluated 160 patients with CMTX1 (87 males and 73 females) from 120 different families who harbored 89 different *GJB1* mutations. Of these 89 mutations, 16 were previously unreported (p.Trp3Leu, p.Asp66His, p.Ile71Thr, p.Arg75Tyr, p.Trp77Arg, p.Val84Asp, p.Tyr135CysfsTer11, p.Arg142Glu, p.Arg142Tyr, p.Arg164Leu, p.Arg164Tyr, p.Thr185Ile, p.Ala196Asp, p.Leu212His, p.Thr269Asn, and p.Asp278_Cys280del). The mutations that are predicted to change the primary amino acid sequence of Cx32 are shown schematically in figure 1.

The 21 most common mutations accounted for 45% of all the identified mutations in families that were not known to be related (in order; p.Arg15Gln, p.Glu102Gly, p.Arg22Ter, p.Ala39Val, p.His126Tyr, p.Arg164Gln, p.Asp278_Cys280del, p.Ser26Leu, p.Ile28Asn, p.Gly159Asp, p.Arg183His, p.Ser182Cys, p.Ala40Val, p.Val63Ile, p.Cys173Arg, p.Arg220Ter, c.-17G>A, c.-103C>T, p.Arg142Trp, c.-17G>A and c.-373G>A). Collectively, the mutations in the 5′ untranslated region were as common as the most common mutation (p.Arg15Gln) in our analysis. The frequencies of the mutations in our cohort are shown graphically in figure e-1 at Neurology.org.

**Figure 2:**
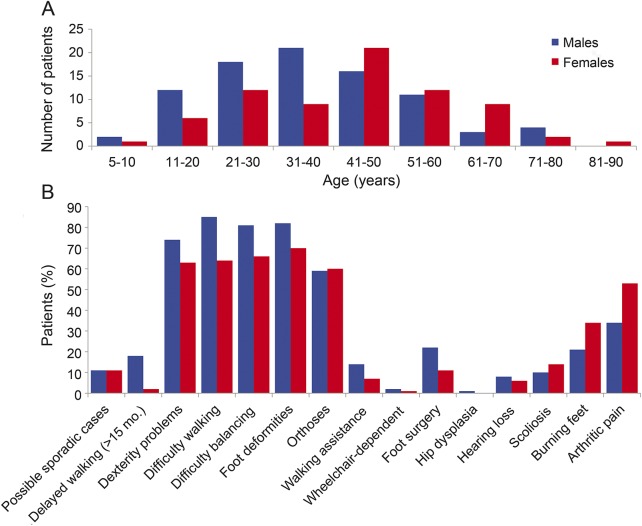
Age, sex, and clinical characteristics of patients with CMTX1 in the cohort (A) Frequency distribution of age and sex of patients with CMTX1 at the age they were first given a CMTNS in an Inherited Neuropathies Consortium clinic. (B) Clinical characteristics separated by sex of 160 patients with CMTX1. CMTX1 = X-linked form of Charcot-Marie-Tooth disease.

Figure 2A represents the distribution of patients according to age and sex at the time they were first given a CMTNS in an INC clinic. The age of patients ranged from 5 to 82 years, with a mean of 43 years. We evaluated 5 members of 1 family, 3 members of 8 families, 2 members of 20 families, and 1 member of 91 families. Of the 91 families with a single evaluated member, 73 had other affected family members who we did not see; the remaining 18 patients had no other known affected family members; we considered these to be possible sporadic cases, and they account for 11% of both male and female patients (figure 2B).

The clinical characteristics of our cohort are shown in figure 2B. When comparing clinical deficits between women and men, the older age distribution of women should be kept in mind (their deficits would be expected to worsen with age), in addition to the consequences of their mosaicism for the expression of the mutant *GJB1* allele (this would be expected to ameliorate their deficits). Delayed walking after the age of 15 months was reported by 18% of males and 2% of females (*p* = 0.003), and 85% of males reported difficulty with walking compared with 64% of females (*p* = 0.008). The age at onset in difficulty walking was younger in men compared with women (19 and 22 years, respectively; *p* = 0.004). Foot deformities and history of prior foot surgery were not statistically different between men and women (*p* = 0.1 and *p* = 1.0, respectively). Almost all patients were ambulatory, although more than half required orthoses. Walking aids were reported in a minority of patients (14% of males and 7% of females), and few (2% of males and 1% of females) required the use of a wheelchair for ambulation. Although males were more likely to report difficulties with balance and dexterity in the hands, these differences were not statistically significant. Partial hearing loss was reported in 7% of patients, but outcomes of formal hearing tests were not available to us—they harbored c.-17G>A, p.Phe51Leu, p.Ile71Thr, p.His94Tyr, p.Glu102Gly, p.His126Tyr, p.Arg142Trp, p.Arg164Gln, p.Cys179Tyr, and p.Thr191Phe193Ter mutations. One male patient (p.Leu165Pro mutation) was noted to have hip dysplasia. Optic nerve atrophy was not noted in any patient by routine funduscopic examination. Scoliosis was reported or noted in 10% of male patients and 14% of female patients (*p* = 0.5; p.Leu9Trp, p.Val13Met, p.Arg22Ter, p.Trp24Cys, p.Ala40Val, p.Ser42fs, p.Thr55Arg, p.Arg75Tyr, p.Val91Gly, p.His94Tyr, p.His126Tyr, p.Arg142Tyr, p.Arg142Trp, p.Cys173Arg, p.Cys179Tyr, p.Arg264Cys, p.Thr269Asn, and p.Ter284Ser mutations). Female patients were more likely than males to report arthritic pain that was present most of the time (*p* = 0.03). Female patients were also more likely than males to report paresthesias with burning features, 34% and 21%, respectively, although this difference was not statistically significant (table 1 and table e-1).

**Table 1:**
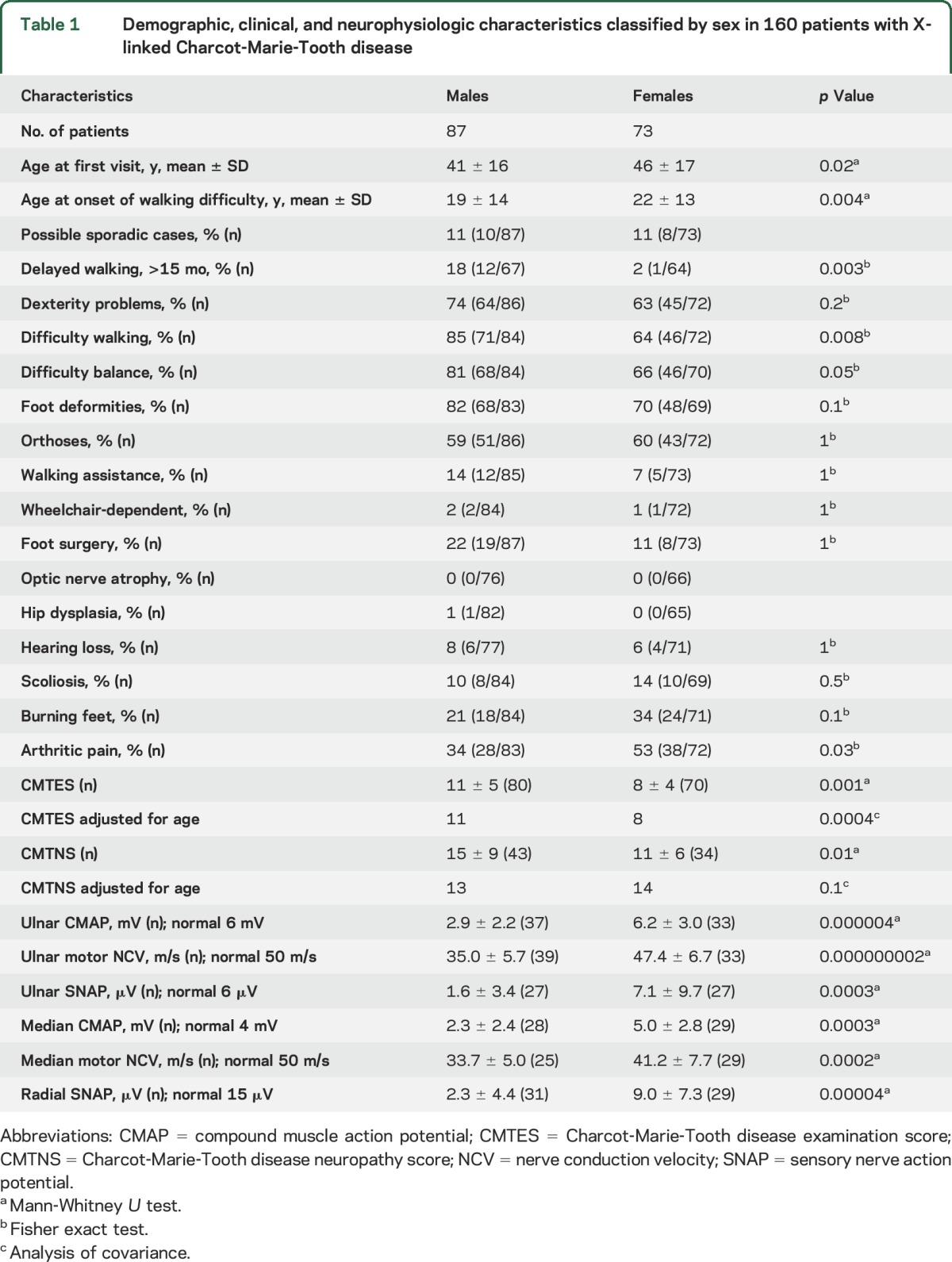
Demographic, clinical, and neurophysiologic characteristics classified by sex in 160 patients with X-linked Charcot-Marie-Tooth disease

At their initial visit, males with CMTX1 were more affected clinically as identified through the CMTES (mean score = 11) compared with the CMTES reported for females with CMTX1 (mean score = 8, *p* = 0.001). Adjusting for age improved the statistical power without affecting the difference in mean CMTES scores between men and women. The combined clinical and neurophysiologic assessments, the CMTNS, showed a similar trend, in which males were more severely affected than females (15 and 11, respectively, *p* = 0.01). Correcting for the age difference between men and women abolished the statistical difference in CMTNS (table 1).

**Figure 3:**
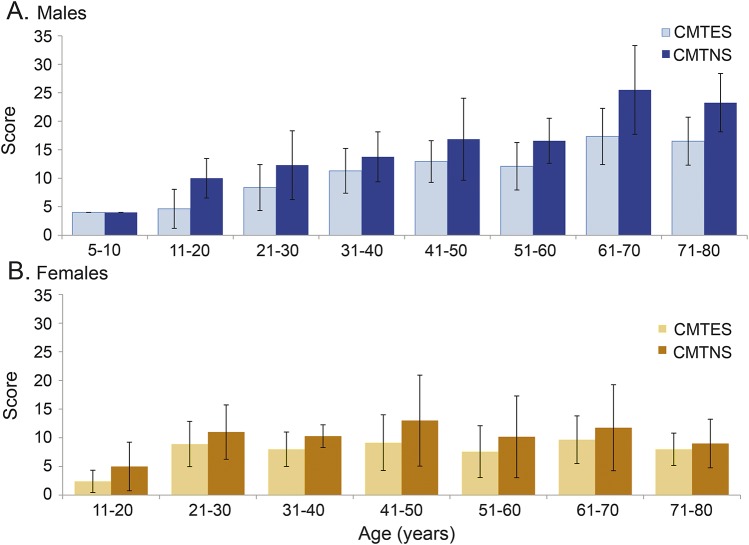
CMT examination score and CMT neuropathy score in male patients with CMTX1 (A) CMTES and CMTNS in male patients with CMTX1 grouped by age. The CMTES is based on history and neurologic examination; the CMTNS adds neurophysiology. Both CMTES and CMTNS increase with age in male patients with CMTX1 (*p* < 0.0005 for both CMTES and CMTNS, analysis of variance). (B) CMTES and CMTNS in female patients with CMTX1 grouped by age. There is an increase in CMTES (but not CMTNS) after the second decade (*p* < 0.03, analysis of variance). CMTES = Charcot-Marie-Tooth disease examination score; CMTNS = Charcot-Marie-Tooth disease neuropathy score; CMTX1 = X-linked form of Charcot-Marie-Tooth disease.

We observed a strong correlation between disability and age when male patients were stratified into groups by age in decades (figure 3A). Increasing age was strongly associated with increasing CMTES and CMTNS scores in men, indicating increased disability in male patients with CMTX1 (*p* < 0.0001) (figure e-2). An analysis of CMTES and CMTNS scores of female patients stratified into groups by age showed a change in CMTES, but not CMTNS, for females with CMTX1 (*p* < 0.03). There was a significant increase in CMTES before the third decade of life in females that did not change thereafter in a statistically meaningful manner (figure 3B). Contrary to men with CMTX1, there was no correlation between advancing age throughout life (as opposed to when stratified by age group) and CMTES or CMTNS in females (figure e-2).

**Figure 4:**
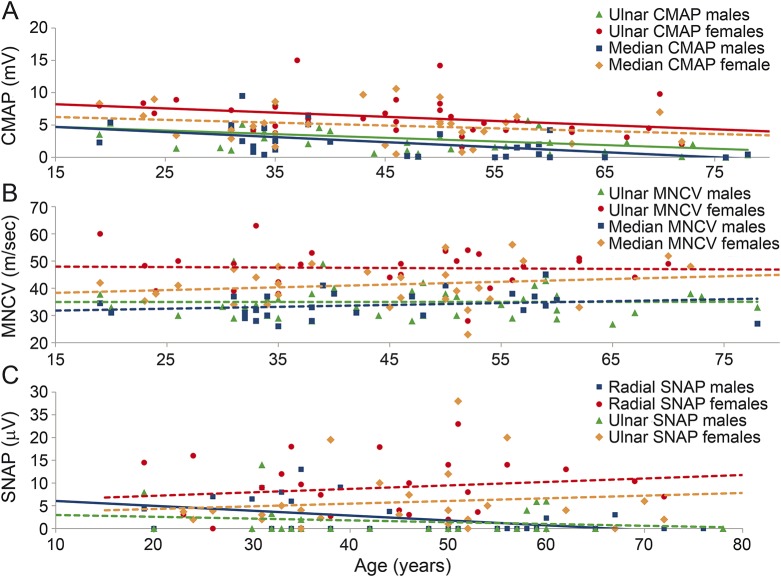
Nerve conduction studies in male and female patients with CMTX1 according to age (A) Ulnar and median nerve CMAPs in males and females with CMTX1 by age. The data from individual patients are represented as single points. There is a correlation between the ulnar and median CMAP with age in males (*p* = 0.02, Spearman ρ = −0.4, and *p* = 0.002, Spearman ρ = −0.6, respectively) and ulnar CMAP with age in females (*p* = 0.002, Spearman ρ = −0.6). A correlation is not observed between median CMAP with age in females (*p* = 0.3, Spearman ρ = −0.2). (B) Ulnar and median motor NCVs in males and females with CMTX1 by age. The data from individual patients are represented as single points. Nerves that did not have a recordable response were excluded. A correlation is not observed between ulnar and median motor NCVs in males with age (*p* = 0.8 and 0.2, Spearman ρ = 0.04 and 0.30, respectively). A correlation is also not observed between ulnar and median motor NCVs in females with age (*p* = 0.7 and 0.4, Spearman ρ = 0.06 and 0.10, respectively). (C) Amplitude of the ulnar and radial SNAPs in males and females with CMTX1 by age. The data from individual patients are represented as single points. There is a correlation between the radial sensory SNAPs with age in males (*p* = 0.01, Spearman ρ = −0.4). A correlation is not observed between ulnar sensory SNAPs with age in males (*p* = 0.6, Spearman ρ = −0.1). A correlation is also not observed between ulnar and radial SNAPs with age in females (*p* = 0.8 and 0.9, Spearman ρ = 0.04 and 0.01, respectively). CMAP = compound muscle action potential; CMTX1 = X-linked form of Charcot-Marie-Tooth disease; MNCV = motor nerve conduction velocity; SNAP = sensory nerve action potential.

In men, the median compound muscle action potential (CMAP) was reduced compared with women (2.5 ± 2.4 vs 4.9 ± 2.9 mV; *p* = 0.001), and we observed a declining correlation with age in men (*p* = 0.01, ρ = −0.5) but not in women (figure 4A). The ulnar CMAP was also smaller in men than in women (3.6 ± 2.6 vs 6.2 ± 3.0; *p* = 0.0004), and we observed a declining correlation with age in women (*p* = 0.04, ρ = −0.36) but not in men (figure 4A). In accord, we found that the ulnar motor nerve conduction velocity (NCV) was slower in male patients than in female patients (35.4 ± 5.8 vs 47.4 ± 6.7 m/s; *p* < 0.0005). Men also had a slower median motor NCV (33.7 ± 5.0 vs 41.2 ± 7.7; *p* = 0.004). The median and ulnar motor NCVs did not slow considerably with age in either males or females (figure 4B). The amplitudes of the ulnar (1.7 ± 3.5 vs 7.1 ± 9.7; *p* = 0.0007) and radial sensory nerve action potentials (SNAPs) (3.5 ± 4.4 vs 9.3 ± 7.1; *p* = 0.0004) were lower in men than in women (figure 4C). These neurophysiologic assessments for men and women are represented in table 1.

We also analyzed missense mutations in 9 domains of Cx32—the 4 transmembrane domains, 2 extracellular loops, a cytoplasmic loop, and cytoplasmic N- and C-termini, in relation to age-adjusted CMTES in male patients to assess for phenotypic severity. Although missense mutations in the transmembrane domains and the 2 extracellular loops were associated with higher disease burden compared with mutations in the cytoplasmic and terminal domains of the protein, the differences were not statistically significant in ANCOVA with age used as a covariant (figure e-3). A similar ANCOVA using all mutations did not identify a specific mutation causing a milder or more severe phenotype in males or females.

## DISCUSSION

The 160 patients with CMTX1 enrolled in the INC are the largest reported cohort of patients with genetically confirmed CMTX1. We found 89 different *GJB1* mutations, including 18 that have not been previously described. As shown in figure 1, these 89 mutations span all the domains of Cx32, so their associated phenotypes should be representative of people with CMTX1. We did not identify specific mutations that resulted in a milder or more severe phenotype in males or females, consistent with a previous report that men with CMTX1 have a similar phenotype regardless of the mutation.^7^ Although missense mutations in some domains of Cx32 (i.e., transmembrane domains and extracellular loops) were associated with higher CMTES and more severe disease phenotype in males, the differences were not statistically significant, possibly because of the small sample size (61 missense mutations in total) and the weak age covariate that could not compensate for the loss of the error degree of freedom.

As previously reported,^7,14^ men were affected more than women in terms of their age at onset and severity at any given age. The age at onset of walking difficulties was significantly earlier in men than in women (mean age of 19 and 22, respectively), and men presented for evaluation at a younger age (mean age 41) compared with women (mean age 46). Men were also more likely than women to complain of difficulties with walking (85% and 64%, respectively). Men were more likely than women to report delayed walking after the age of 15 months; although a majority reported walking before the age of 15 months (82%). Almost all women in our cohort reported walking before the age of 15 months in comparison (98%). In considering these differences between the sexes, there is a potential of recall bias between men and women in terms of their reporting. Women were more likely than men to have arthritic pain; this was reported in about half of the female patients. The increased risk of arthritic pain in women is consistent with the extensive literature of increased risk of clinical pain and increased sensitivity to chronic pain in women compared with men.^17^ The specific etiologic basis underlying the sex differences related to pain remains elusive.

On the other hand, some phenotypic features of the disease were indistinguishable between men and women. For example, difficulties with hand dexterity and balance, the need for orthoses, scoliosis, and paresthesias in the feet were indistinguishable in their frequency in men and women. There were also no statistical differences between the sexes in terms of their need for walking aids, or foot deformities and surgeries, or wheelchair dependence (which was rare in both sexes; 2% of males and 1% of females).

Consistent with what is known in CMTX1,^7^ male patients in our cohort were more affected both clinically and neurophysiologically than females. The majority of patients had a moderate level of impairment on the CMTNS score on presentation (mean score 13). Male patients had higher CMTES and CMTNS scores (mean CMTNS score 15) compared with females (mean CMTNS score 11) indicating a higher disease burden (table 1). The difference in mean CMTNS between the sexes was more sensitive to statistical correction for age given the smaller sample size and less robust initial statistical significance (table 1). The absence of statistical difference in CMTNS after adjusting for age should not to be interpreted as evidence of comparable disease burden between the sexes. The disease burden, as quantified by CMTES and CMTNS, increased with age in men with CMTX1 in contrast to women, in whom the disease burden remained static after the third decade of life. This static disease burden in females might be due to the fact that the rate of disease progression is below clinical detection after the third decade. Increasing disability with age in male patients with CMTX1 has been recognized in the past.^7^ We observed a difference in disease burden in women as measured by CMTES before the third decade when women were stratified by the age group (figure 3B). These data suggest that although men are affected at an earlier age and are more severely affected than women, the disease presents early in the life of women and particularly before the third decade. The age at onset of walking difficulties in women in our cohort (mean of 22 years) supports this interpretation. We state this with caution, however, given the inherent limitations of extending cross-sectional analysis in predicting disease progression over a lifetime, and the possibility that the less affected woman did not participate in our study.

NCVs have been traditionally used to separate demyelinating and axonal forms of CMT. CMTX1 has been neurophysiologically characterized as having “intermediate” slowing—typically 30–40 m/s in affected males and 30–50 m/s in affected females.^7,10,18^ Compared with females with CMTX1, males had reduced mean ulnar and median CMAPs and NCVs as well as SNAP amplitudes (table 1). We found a decreasing correlation of the median and ulnar nerve CMAP with age in males, whereas there was a decreasing correlation of ulnar nerve CMAP with age in females (figure 4A). Although we did not find statistical correlations between age and median nerve CMAPs in females in our cohort, this should not be interpreted as sparing of the median nerve in females. NCVs were insensitive to advancing age in our study in both sexes. The faster conduction velocity in women likely owes to the lesser degree of demyelination and remyelination in their nerves, the result of Schwann cells that express the wild-type *GJB1* gene. We also found a decreasing correlation of the radial nerve SNAP amplitudes with age in males (figure 4C). We did not find correlations between age and ulnar SNAPs in men and SNAP amplitudes in women. The decreased correlations in CMAP and SNAP amplitudes likely reflect the loss of large myelinated, motor and sensory axons, respectively.

Although no pharmacologic interventions exist for CMTX1, supportive care in the form of gait stabilization, addressing foot drop, and treating neuropathic and mechanical pain are the mainstays of current therapy. Although men have higher disease burden in general, our data support the notion that women are equally likely to report difficulties with balance and problems with hand dexterity. Early recognition of these clinical features might therefore be important in the management of female patients with CMTX1. Recognition of hand dexterity symptoms in CMTX1 and directing patients to appropriate therapies (e.g., occupational therapy) is therefore of paramount importance.^19^ Although females have less overall disease burden compared with males as is evident through CMTNS or CMTES, they are more likely to have arthritic type pain requiring symptomatic therapy. Women with CMTX1 will also likely require the same attention to orthotic therapy as their male counterparts based on the data from this cohort.

With the advent of high-throughput screening for drug candidates in CMT,^20^ preclinical evidence of benefit in immune modulation,^21^ and a successful gene replacement therapy in a mouse model of CMTX1,^22^ one anticipates therapeutic clinical trials in CMTX1. This effort will benefit from detailed disease natural history studies, sensitive and robust outcome measures, and an appropriate patient selection strategy. In the absence of phenotypic correlation with specific *GJB1* mutations, one envisages that patient selection will consider the age and sex as the key considerations. Based on our data, investigating males makes sense: they are clearly affected earlier in life, have more severe disease burden at any given age, and their disability progresses with age, whereas phenotypic disease expression in females is seemingly unaffected by advancing age after the third decade. Having longitudinal data on these points will be key.

## Supplementary Material

Data Supplement

Coinvestigators

## GLOSSARY

ANCOVA: analysis of covariance
CMAP: compound muscle action potential
CMT: Charcot-Marie-Tooth disease
CMTES: Charcot-Marie-Tooth disease examination score
CMTNS: Charcot-Marie-Tooth disease neuropathy score
CMTX1: X-linked form of Charcot-Marie-Tooth disease
Cx32: connexin32
INC: Inherited Neuropathies Consortium
NCV: nerve conduction velocity
SNAP: sensory nerve action potential
