# Supplementary material for: Cross-sectional analysis of a large cohort with X-linked Charcot-Marie-Tooth disease (CMTX1)
Source: Neurology. 2017 Aug 29;89(9):927–35. doi: 10.1212/WNL.0000000000004296 (PMC5577965; doi:10.1212/WNL.0000000000004296)
Supplement: Data Supplement [file supp_89_9_927__index.html]

Cross-sectional analysis of a large cohort with X-linked Charcot-Marie-Tooth disease (CMTX1) — Data Supplement 

# Cross-sectional analysis of a large cohort with X-linked Charcot-Marie-Tooth disease (CMTX1)

## Data Supplement

**Neurology® data supplements are not copyedited before publication. Published editorials and translations have been copyedited.  
 © 2017 American Academy of Neurology.  
  
 Files in this Data Supplement:**

- Data Supplement - Microsoft Word file
