# Supplementary material for: Cross-sectional analysis of a large cohort with X-linked Charcot-Marie-Tooth disease (CMTX1)
Source: Neurology. 2017 Aug 29;89(9):927–35. doi: 10.1212/WNL.0000000000004296 (PMC5577965; doi:10.1212/WNL.0000000000004296)
Supplement: Data Supplement [file supp_WNL.0000000000004296_Supplemental_Data.docx]

# Supplemental Data

Table e-1: Previously unreported *GJB1* mutations in our cohort

| DNA sequence change | Amino acid sequence change |
| --- | --- |
| c.8G>T | p.Trp3Leu |
| c.196G>C | p.Asp66His |
| c.212T>C | p.Ile71Thr |
| c.223C>T | p.Arg75Tyr |
| c.229T>A | p.Trp77Arg |
| c.251T>A | p.Val84Asp |
| c.403_404delTA | p.Tyr135CysfsTer11 |
| c.425G>A | p.Arg142Glu |
| c.486C>T | p.Arg142Tyr |
| NA | p.Arg164Leu |
| c.552C>T | p.Arg164Tyr |
| c.554C>T | p.Thr185Ile |
| c.587C>A | p.Ala196Asp |
| c.635T>A | p.Leu212His |
| c.806C>A and c.851G>C | p.Thr269Asn and p.Ter284Ser |
| c.832_840del | p.Asp278_Cys280del |


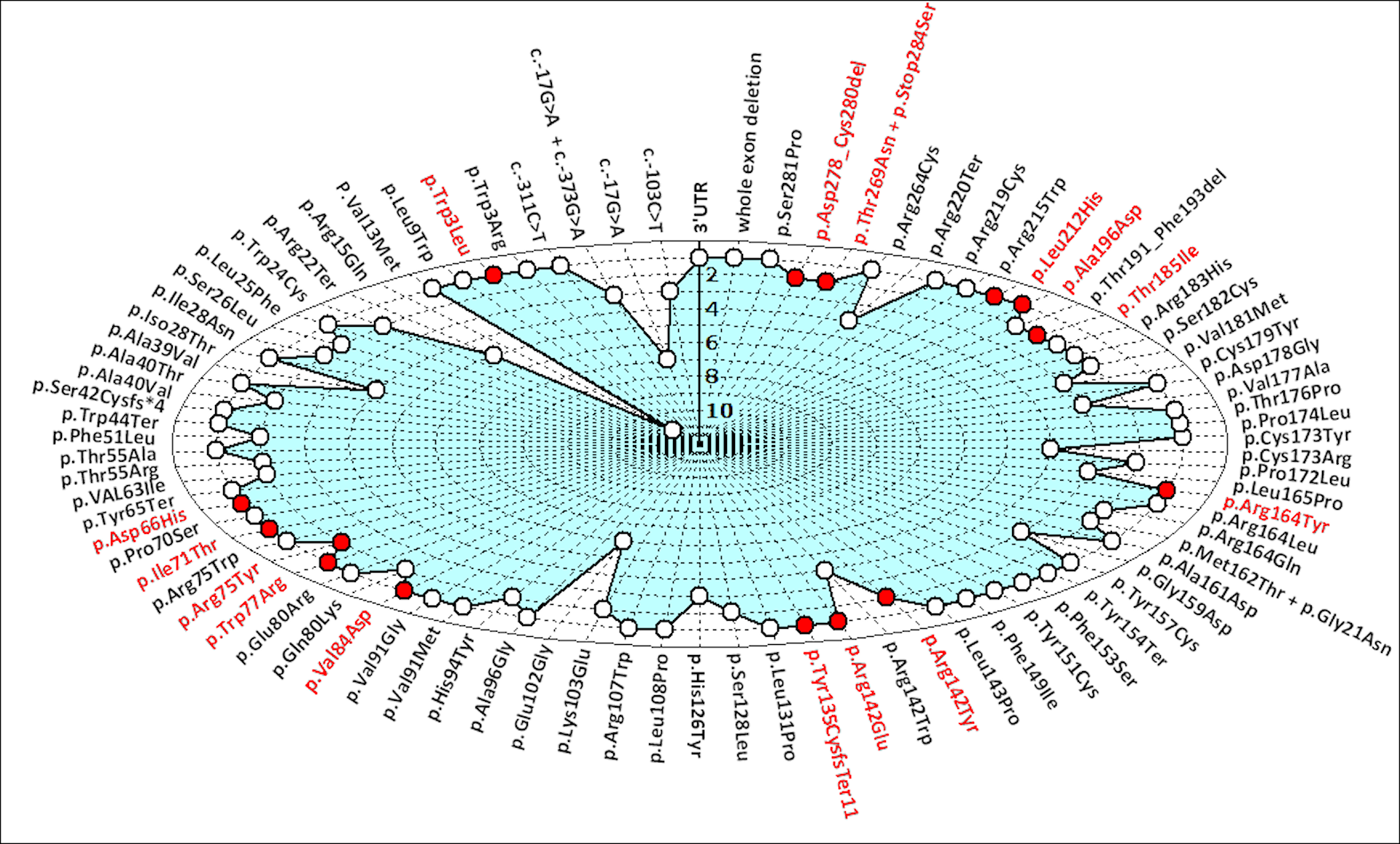


Figure e-1: A plot demonstrating the number of patients with specific *GJB1* mutations in our reported cohort. Previously unreported mutations are marked in red.


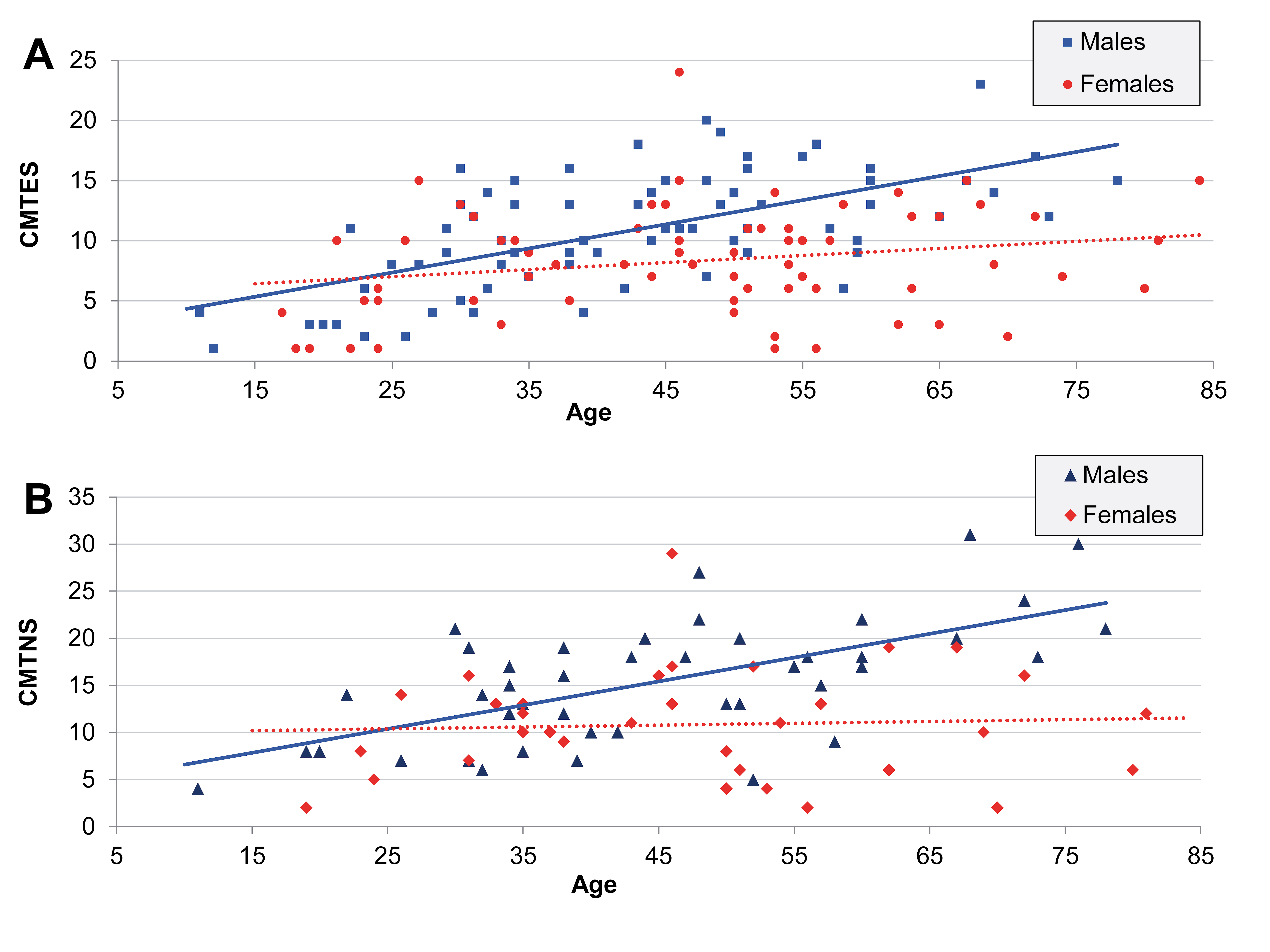


Figure e-2: (A) The CMT Examination Score (CMTES) in males and females with CMTX1 by age. The CMTES is based on history and neurological examination. CMTES increases with age in males (p<0.0001, Spearman’s rho 0.59), but not in females (p=0.1, Spearman’s rho 0.20). (B) The CMT Neuropathy Score (CMTNS) in males and females with CMTX1 by age. The CMTNS adds neurophysiology into the CMTES. CMTNS increases with age in males (p<0.0001, Spearman’s rho 0.57), but not in females (p=0.9, Spearman’s rho 0.02).


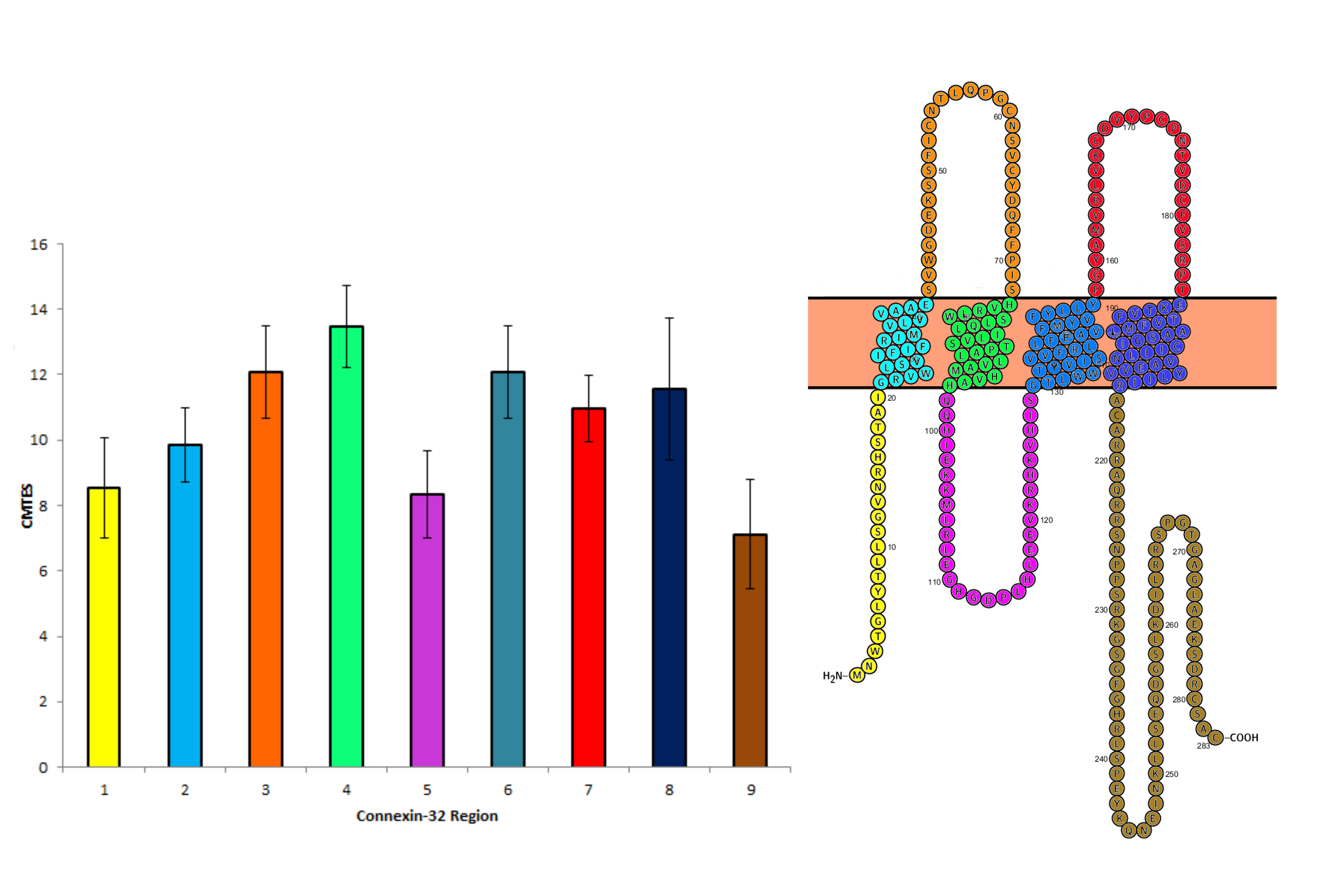


Figure e-3: CMTES score in men (adjusted for age) in relation to location of their missense mutation in Cx32. Mutations in four transmembrane domains (represented by regions 2, 4, 6 and 8), two extracellular loops (regions 3 and 7), a cytoplasmic loop (region 5) and cytoplasmic N- and C-terminal domains (regions 1 and 9 respectively), were compared in terms of age adjusted CMTES. Although missense mutations in transmembrane domains and extracellular loops were associated with higher disease burden in comparison to mutations in the cytoplasmic and terminal domains of the protein, the differences were not statistically significant (p=0.1, ANCOVA). A reported crystal structure of Cx26 [^26^](#_ENREF_26) was used in predicting the domains of Cx32 for this analysis.
