# Supplementary material for: Cross-sectional analysis of a large cohort with X-linked Charcot-Marie-Tooth disease (CMTX1)
Source: Neurology. 2017 Aug 29;89(9):927–35. doi: 10.1212/WNL.0000000000004296 (PMC5577965; doi:10.1212/WNL.0000000000004296)
Supplement: Coinvestigators [file supp_WNL.0000000000004296_Coinvestigators-Inherited_Neuropathies_Consortium.docx]

Inherited Neuropathies Consortium (INC)

Richard Lewis, MD (Cedars-Sinai Medical Center, Principal Site Investigator)

John Day, MD, PhD (Stanford University, Principal Site Investigator)

Gyula Acsadi, MD, PhD (Connecticut Children’s Medical Center, Principal Site Investigator)

Mario Saporta, MD, PhD (University of Miami, Principal Site Investigator)

Stephan Züchner, MD (University of Miami HIGH Biorepository, Principal Site Investigator)

Richard Finkel, MD (Nemours Children's Clinic, Principal Site Investigator)

Michael Shy, MD (University of Iowa, Principal Site Investigator, INC Director)

Shawna Feely, MS, CGC (University of Iowa, Consortium Project Manager)

Thomas Lloyd MD, PhD (Johns Hopkins University, Principal Site Investigator)

Vera Fridman, MD (Massachusetts General Hospital, Principal Site Investigator)

Sindhu Ramchandren, MD, MS (University of Michigan, Principal Site Investigator)

David Walk, MD (University of Minnesota, Principal Site Investigator)

David Herrmann, MBBCh, (University of Rochester, Principal Site Investigator)

Erig Logigian, MD (University of Rochester, Site Co-Investigator)

Michael Stanton, MD (University of Rochester, Site Co-Investigator)

Katy Eichinger, PT, DPT, NCS. (University of Rochester, Sub-Investigator)

Debra Guntrum, MS, FNP. (University of Rochester, Sub-Investigator)

Cindy Gibson, MS, NP. (University of Rochester, Sub-Investigator)

Steven Scherer, MD, PhD (University of Pennsylvania School of Medicine, Principle Site Investigator)

Sabrina Yum, MD (Children's Hospital of Philadelphia, Principle Site Investigator)

Jun Li, MD, PhD (Vanderbilt University, Principle Site Investigator)

Joshua Burns, Ph.D. (The Children's Hospital at Westmead, Principle Site Investigator)

Davide Pareyson, MD (C. Fondazione IRCCS Istituto Neurologico Carlo Besta, Principle Site Investigator)

Isabella Moroni, MD (C. Fondazione IRCCS Istituto Neurologico Carlo Besta, Site Co-Investigator)

Chiara Pisciotta, MD, PhD (C. Fondazione IRCCS Istituto Neurologico Carlo Besta, Site Sub-Investigator)

Mary Reilly, MD (UCL, National Hospital for Neurology and Neurosurgery, Principle Site Investigator)

Matilde Laurá, MD (UCL, National Hospital for Neurology and Neurosurgery, Site Co-Investigator)

Francesco Muntoni (Great Ormond St Hospital for Children, Principle Site Investigator)
